# miRNA Regulatory Circuits in ES Cells Differentiation: A Chemical Kinetics Modeling Approach

**DOI:** 10.1371/journal.pone.0023263

**Published:** 2011-10-19

**Authors:** Zijun Luo, Xuping Xu, Peili Gu, David Lonard, Preethi H. Gunaratne, Austin J. Cooney, Robert Azencott

**Affiliations:** 1 Department of Mathematics, University of Houston, Houston, Texas, United States of America; 2 Department of Molecular and Cellular Biology, Baylor College of Medicine, Houston, Texas, United States of America; 3 Biology and Biochemistry Department, University of Houston, Houston, Texas, United States of America; University of South Florida College of Medicine, United States of America

## Abstract

MicroRNAs (miRNAs) play an important role in gene regulation for Embryonic Stem cells (ES cells), where they either down-regulate target mRNA genes by degradation or repress protein expression of these mRNA genes by inhibiting translation. Well known tables TargetScan and miRanda may predict quite long lists of potential miRNAs inhibitors for each mRNA gene, and one of our goals was to strongly narrow down the list of mRNA targets potentially repressed by a known large list of 400 miRNAs. Our paper focuses on algorithmic analysis of ES cells microarray data to reliably detect repressive interactions between miRNAs and mRNAs. We model, by chemical kinetics equations, the interaction architectures implementing the two basic silencing processes of miRNAs, namely “direct degradation” or “translation inhibition” of targeted mRNAs. For each pair (M,G) of potentially interacting miRMA gene M and mRNA gene G, we parameterize our associated kinetic equations by optimizing their fit with microarray data. When this fit is high enough, we validate the pair (M,G) as a highly probable repressive interaction. This approach leads to the computation of a highly selective and drastically reduced list of repressive pairs (M,G) involved in ES cells differentiation.

## Introduction

MicroRNAs (miRNAs) are small non-coding RNAs, 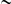
22 nucleotides in length that are able to bind and repress protein coding mRNAs through complementary base pairing. The minimum requirement for this interaction is six consecutive nucleotides, which undergo base pairing to establish a miRNA-mRNA duplex. The only constraints being that the six nucleotides must be localized in the 5′seed sequence (between nucleotides 2–8) of the miRNA and the complementary binding sites, which are largely located in the 3′-untranslated regions (3′-UTRs) of target mRNAs.

Because of this very minimal binding requirement, a given miRNA can potentially bind and silence hundreds of mRNAs across a number of signaling pathways to integrate multiple genes into biologically meaningful networks regulating a variety of cellular processes [1]–[3]. In animals, miRNAs regulate gene expression post-transcriptionally by either down-regulating their target mRNAs or by inhibiting their translation [4]. MiRNAs have two types of effects on their target mRNAs. When a miRNA M binds to its target mRNA gene G with partial complementarity, then the translation of gene G is inhibited; however, when M binds to its target G with near-perfect complementarity, then gene G is cleaved, resulting in its degradation. Thus, when we ectopically over-express a miRNA we expect to see a decrease in the target genes at the protein level but not at the gene level if the miRNA-mRNA duplex is formed through imperfect complementarity. In contrast, we expect both mRNA and protein levels to change when the miRNA-mRNA duplex binds with near perfect complementarity.

Expression of miRNA genes is ultimately controlled by the same transcription factors which regulate the expression of protein coding genes. The expression of these same transcription factors can in turn be regulated by miRNAs, leading to positive and negative feedback loops [5]–[7]. Thus transcription factors such as Oct4, Sox2 and Nanog, which regulate gene networks controlling key properties of ES cells, are closely linked with miRNAs that are enriched in ES cells in both mice and humans [5], [8], [9].

Genome-wide studies using microarray and sequencing technologies have significantly expanded our knowledge of the complex regulatory networks underpinning the key properties of ES cells, namely self-renewal and pluripotency. Classical methods like sequence analysis, correlation analysis and other statistical inference techniques, have often yielded very large lists of potentially interacting miRNA-mRNA pairs, so that experimental testing of all possible interactions would be too costly.

In previous work on ES cells regulatory network, ES cells microarray data recorded during differentiation were mainly studied by linear correlation analysis, focused on simultaneity of high miRNA levels and low mRNA levels or vise versa. But correlation analysis cannot tell whether miRNAs and their target genes/proteins interact directly or indirectly, nor give clear indication about the interaction mechanisms.

In this paper, we deepen the analysis of several ES cells microarray data, by parameterized chemical kinetics modeling of miRNA-mRNA interactions, involving associated protein products. Our goal was to drastically narrow down the list of potential repressive miRNA-mRNA links. We define two specific chemical kinetic models underlying the two basic repressive actions of a typical miRNA on a targeted mRNA gene G, namely by direct degrading of G or by inhibiting the translation of the protein generated by G.

We implement fast parameter estimation algorithms to adequately fit these chemical kinetics models to microarray data from ES cells undergoing retinoic acid (RA) induced differentiation and compute a precise *quality of fit* between models and data. We have thus generated, parameterized, and tested more than 10,000 models, to evaluate as many potential instances of miRNA-mRNA interactions. By thresholding the “quality of fit” of these models, we then accept or reject the validity of the associated miRNA-mRNA interaction.

Our presentation here is focused on 10 key regulatory genes for ES cells differentiation, namely Oct4, Nanog, Sox2, Klf4, Esrrb, cMyc, Tbx3, Ezh1, Ezh2, Eed, and on the main miRNAs which may target these 10 key genes, according to the *in silico* target prediction databases TargetScan (version 5.0) and/or miRanda. Our approach radically narrows down the lists of potentially interacting miRNA-mRNA pairs predicted by TargetScan or miRanda, and for each validated miRNA-mRNA pair, we identify wether target mRNA repression occurs by direct degradation or by translation inhibition.

## Materials and Methods

### Microarray Data Description

We have centered our miRNA-mRNA interactions study on microarray data of mouse ES cells undergoing RA-induced differentiation. This dataset had been previously analyzed by classical techniques in Gu et al. [5]. The miRNAS microarray was provided by LC Science Inc. Each microarray data file gathers genes expressions recordings from two ES cells differentiation experiments: Wild Type (WT) and GCNF- Knock-Out (KO).

In both experiments, expression levels were recorded for 266 well characterized miRNAs on days 0, 1, 3, 6, based on 6 probe replicates for each miRNA prediction (MCEMIR, Cand, MIR) and 8 probe replicates for miRNAs (mmu-miRs). Simultaneously, the expression profiles of 30,000 mRNAs were recorded on days 0, 3, 6 using an Affymetrix mouse 430 2 array, based on three biological replicates per time point. Expression levels range from 1 to 46,559 for the miRNAs, and from 1 to 21,845 for mRNAs.

For both WT and KO data, and each day, we have several arrays (chips) recording expression levels for our 266 miRNAs and 30,000 mRNAs. For each miRNA and each mRNA, and for each day, we synthesize the replicate recordings by simply averaging the available multiple values of their expression levels.

For each miRNA and each mRNA, we can then interpolate the available profile data to generate interpolated expression levels values at 19 intermediary time points (t = 0, 1/3, 2/3, …, 17/3, 18/3), by a Piecewise Cubic Hermite Interpolation (PCHIP) technique, which is well known to preserve monotonicity and the basic qualitative features of expression profiles.

### Analysis of Western Blot Data for Four Pluripotency Proteins

**Figure 1:**
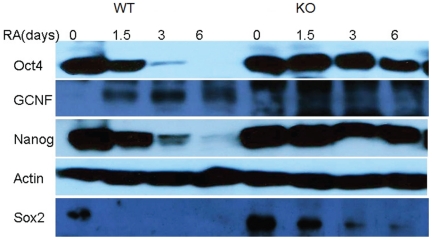
Western blots for 4 proteins and actins. Oct4 and Nanog levels exhibit quite strong decrease for WT cells and very slow decrease for GCNF-KO cells. Sox2 levels vanish after 1.5 days for WT cells and slowly decrease for GCNF-KO cells. GCNF levels are initially low, peak at day 3 and fall back on day 6 for WT cells. For GCNF-KO cells, GCNF levels naturally vanish.

By Western blots analysis, we have also recorded protein expression profiles For 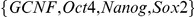 during ES cell differentiation (see Figure 1), for both WT and GCNF-KO, at time points (0, 1.5, 3, 6).

The raw image data provided by Western blotting were converted into numerical values by standard image analysis software tools [10]. We have then normalized the image intensities by the corresponding recorded actin intensity (which functions as an internal loading control). These normalized proteins intensity data were then interpolated as above to compute proteins expressions at the same 19 intermediary time points.

### Previous Results Linking miRNAs and Regulatory Loops for ES Cells Differentiation

Several publications indicate that miRNAs have important functions in post-transcriptional silencing and are involved in the regulation of self-renewal and of differentiation for ES cells. In the ES cells differentiation study [5], the authors classified miRNAs into three classes: for class HL, expression levels are High on days 0–1 and Low on day 6; for class LH, expression levels are Low on days 0–1 and High on day 6; class TR gathers all other “Transient” expression profiles.

Our microarray recordings included 46 miRNAs in class TR, 105 miRNAs in class HL, and 78 miRNAs in class LH. According to TargetScan and/or miRanda, pluripotency regulatory genes in ES cells are targeted by 26 of the 105 miRNAs in class HL, and by 23 of the 78 miRNAs in class LH.

**Figure 2:**
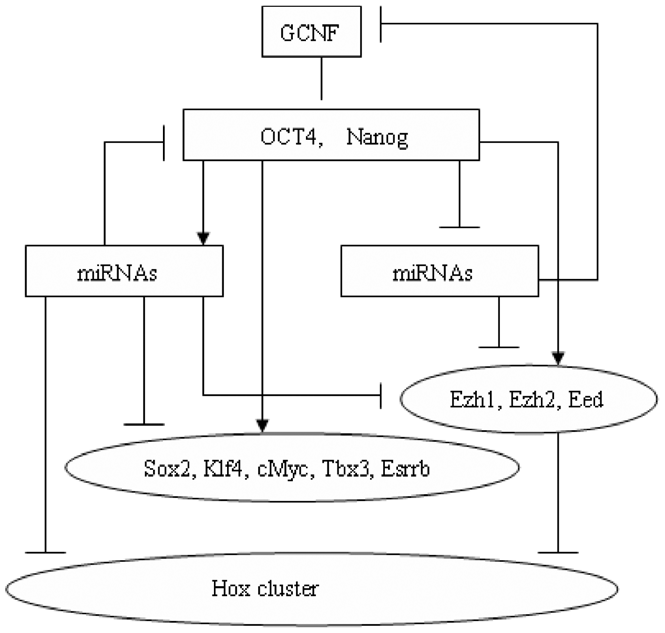
A few key regulatory loops for ES cells according to [**5**]. Arrows indicate “activation” while bars ending with a hash indicate “repression”.

To detect interacting miRNA-mRNA pairs [5], used qualitative correlation of expression profiles, mostly for miRMNA classes HL and LH, without any conclusions for miRNA class TR. For Wild Type ES cells differentiation [5], outlined a regulatory network (see Figure 2 [5]) involving the orphan nuclear receptor GCNF (NR6A1), which is a transcriptional repressor of Oct4 and Nanog. Both Oct4 protein and Nanog protein are transcriptional regulators for two groups of mRNAs: the Self-Renewal Regulators SRR (Sox2, Klf4, Esrrb, Tbx3, cMyc), and the Differentiation Inhibitors DI such as the Polycomb complex (Ezh1, Ezh2, Eed). In the Figure 2 network, the miRNAs of class HL target 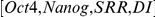 and the Hox cluster, while miRNAs of class LH target 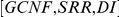.

Our goal was to deepen this analysis, by nonlinear modeling techniques, to validate more precisely if any miRNA “M” actually represses an ES cell regulatory gene G belonging to the list TARG(M) of all mRNAs targeted by M according to miRanda or to TargetScan 5.0. Our approach was to first select several basic small interaction motifs including the pair (M,G), and then to parametrize a chemical kinetic model for each such motif, in order to fit the expression profiles recorded in our microarray data sets. We now introduce the two basic interaction architectures we have systematically modeled.

### Transcription-Degradation (Transcr.Degr.) Architecture Linking miRNAs to ES Cells Regulatory Networks

#### Basic Transcription-Degradation architecture

Our first basic interaction architecture for any miRNA-mRNA pair (M,G) deals with situations where the miRNA “M” directly degrades the transcription of its mRNA target G by direct binding with perfect or near-perfect complementarity. For a fixed ‘downstream” mRNA G, we assume that few miRNAs may *simultaneously* bind with G with near-perfect complementarity.

**Figure 3:**
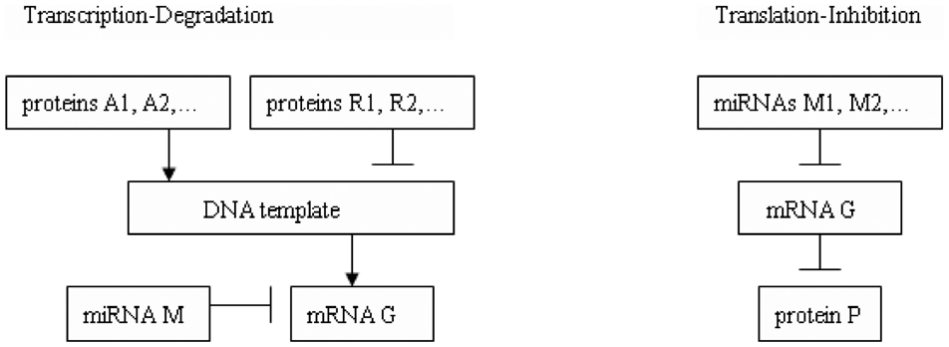
Transcription-Degradation (Transcr.Degr.) architectures and Translation-Inhibition (Transl.Inhib.) architectures.

Hence for each key ES regulatory gene G, we have selected a family *Transcr.Degr(G)* of small *Transcription-Degradation (Transcr.Degr.) architectures* (see Figure 3) potentially involving transcription-degradation of G by one or several miRNAs 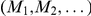 as well as the interactions of G with the main proteins acting as transcriptional factors of G. Combinatorial considerations show that the size of Transcr.Degr.(G) can be quite large (see below).

Here is an example of a small network of Transcr.Degr. type, involving 5 molecules: GCNF, mRNA gene Oct4, Oct4 protein, Nanog protein, and miRNA mmu-miR-186.

Indeed, in view of Figure 2, GCNF is a transcriptional repressor of the mRNA gene Oct4, and miRNAs of class HL may target and degrade Oct4. According to [8], [11], the Oct4 protein and the Nanog protein are potential transcriptional activators of gene Oct4. Finally, by miRanda, the miRNA mmu-miR-186 may target Oct4.

#### Key Families of small networks of Transcr.Degr. type

We now construct the family of all small networks of Transcr.Degr. type involving arbitrary downregulating pairs (M,G) where M is any one of our 266 miRNAs and G is any one of the 10 key ES regulatory factors Oct4, Nanog, Sox2, Klf4, Esrrb, Tbx3, cMyc, Ezh1, Ezh2, Eed. Naturally we require the mRNA gene G to belong to the target list TARG(M), which reduces the initial set of 2660 pairs (M,G) to only 238 pairs where M may target G, including for instance 19 pairs (miRNA, Oct4), 2 pairs (miRNA, Nanog), 29 pairs (miRNA, Sox2), etc.

Based on [5], [11], transcription of Oct4 and Nanog is repressed by GCNF, and activated by Oct4, Nanog, Sox2. So for each one of the 19 potentially downregulating pairs (M,Oct4), we study seven Transcr.Degr. architectures, “combining the molecules M and Oct4 with each one of the following 7 proteins combinations: (Oct4), (Nanog), (Sox2), (Oct4,Nanog), (Oct4,Sox2), (Nanog, Sox2), (Oct4, Nanog, Sox2). This generates 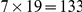 potential networks of Transcr.Degr. type downregulating Oct4 via an miRNA.

By a similar construction, the only 2 pairs (M, Nanog) retained above are associated to 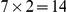 networks of Transcr.Degr. type potentially downregulating Nanog via an miRNA.

When the downstream gene G is in the list L = (Sox2, Klf4, Esrrb, Tbx3, cMyc, Ezh1, Ezh2, Eed) [5], suggests, as seen in Figure 2), that Oct4 and Nanog are transcriptional activators or repressors of G. For each one of the 217 pairs (M,G) retained above with gene G belonging to the list L, we then generate one network of Transcr.Degr. type including M,G, as well as the Oct4 and Nanog proteins. This defines 217 corresponding networks of Transcr.Degr. type.

Thus we have determined a set of 217+147 = 364 potential Transcr.Degr. architectures to be studied below by chemical kinetics model fitting.

### Translation-Inhibition Architectures (Transl.Inhib.) Linking miRMNAs to key ES Cell Regulatory genes

#### Basic Translation-Inhibition Architecture

Our second basic interaction architecture for any miRNA-mRNA pair (M,G) models the cases where the upstream miRNA “M” inhibits the translation of the downstream mRNA gene G, and thus represses the expression of the protein P generated by G. For a fixed mRNA G, we may have several upstream mRNAs 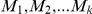 inhibiting the translation of G. The molecules 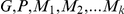 then define a Translation-Inhibition (Transl.Inhib.) architecture (Figure 3).

We have generated Western blot data for the 4 proteins GCNF, Oct4, Nanog, Sox2. Hence in this paper we restrict the study of Transl.Inhib. architectures to the cases where P is one of these 4 proteins. For instance among the 19 miRNAs M potentially targeting gene Oct4, as listed by miRanda or Targetscan, we can select the 3 miRNAs (mmu-mir-290, mmu-mir-296, mmu-mir-138) and study the Transl.Inhib. architecture defined by (gene Oct4, protein Oct4) and these 3 mRNAs.

#### Key Families of small gene networks of Transl.Inhib. type

To restrict somewhat the combinatorial complexity, we have limited to 3 the number of upstream miRNAs involved in any Transl.Inhib. architecture repressing each one of the 3 proteins Oct4, Nanog, Sox2. For the Transl.Inhib. architectures repressing gene GCNF, we have restricted the number of upstream miRNAs to 1. Indeed, since GCNF is not expressed in the KO context, the number of profile points available from our WT microarray is too small to correctly parametrize the Transl.Inhib. architectures repressing GCNF as soom as they involve 2 or more upstream miRNAs.

With these restrictions we have thus defined and studied 5337 Transl.Inhib. architectures potentially repressing one of the 4 proteins GCNF, Oct4, Nanog, Sox2.

### Chemical Kinetic Equations for Transcr.Degr. and Transl.Inhib. Architectures

Select any miRNA-mRNA pair (M,G). Call P the protein generated by gene G. The two main modalities of interaction within the triplet of molecules [G, P, M] were formalized above by the Transcr.Degr. and the Transl.Inhib. architectures. We now model these two types of interactions by chemical kinetic equations linking the expression levels of these molecules.

We point out an essential mathematical property of the two chemical kinetic models introduced below. Under arbitrary scale changes affecting the numerical expression levels of mRNAs, proteins and miRNAs, the chemical kinetic equations for both architectures Transcr.Degr. and Transl.Inhib. will still keep the same mathematical form but with corresponding nonlinear changes in the equations parameters. This is a crucial “model invariance” property for model fitting since raw microarray data and Western blot data are at best roughly proportional to the absolute expression levels of the molecules of interest, and the corresponding constants of proportionality are fundamentally unknown.

#### Chemical Kinetics for Transcr.Degr. Architectures

The transcription-degradation architecture Transcr.Degr. is a small size interaction model formalizing how the rate of change for the expression of a downstream mRNA “G” depends on the expression levels of its main upstream factors. These factors include the post-transcriptional repressor miRNA “M”, and two sets of proteins: the set 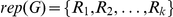 of transcriptional repressors for G, and the set 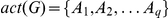 of transcriptional activators for G.

Denote by 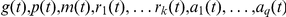 the expression levels of molecules 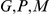, 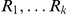, 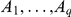 at time 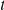.

To model the transcription of downstream mRNA gene G by interaction with transcription repressors rep(G) and activators act(G), we introduce a nonlinear chemical kinetic equation (CKE) similar to equations proposed in [12], [13], but with a complementary term encoding the repressive influence of miRNA M on its target mRNA G, as follows (see [14]).

We first define the individual impacts of proteinic repressors 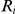 and activators 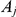 on the rate of change 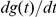 by
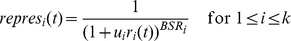
(1)

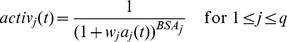
(2)where 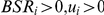 and 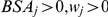 are respectively the number of binding sites and the affinity constants with G for the transcriptional factors 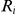 and 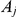.

The synthetic impacts REP(t) and ACT(t) of repressors rep(G) and activators act(G) on 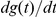 are then (see [12]–[14]) given by
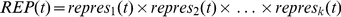
(3)

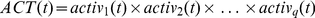
(4)By a probabilistic analysis detailed in [12]–[14], *the fraction* 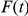 *of DNA templates committed to the transcription of G* can then be estimated by
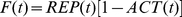
(5)Let 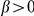 be the degradation rate of G, 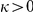 the transcription rate of G, and 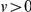 be the reaction rate between G and M. During the small time interval 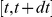, the concentration of new 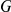 molecules synthesized by transcription is equal to 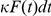, the repressive interaction of molecules 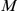 and 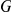 eliminates 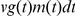 molecules of 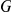, and natural decay destructs 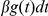 molecules of 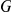. Hence the expression level 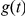 of G verifies the following CKE, characteristic of Transcr.Degr. architectures,
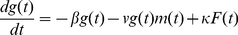
(6)Note that this CKE is parametrized by the 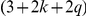 unknown positive parameters 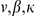, 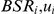, 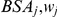.

#### Chemical Kinetics for Transl.Inhib. Architectures

The translation-inhibition architectures Transl.Inhib. involves one downstream mRNA gene G, the protein P produced by G, and a selected set MIR(G) of n upstream miRNAs 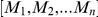 repressing the translation of G. The concentrations at time 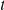 of 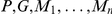, are denoted by 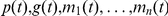. We have modeled Transl.Inhib. architecture by the following chemical kinetics equation [12], [14]. For each miRNA 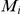, call 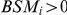 the number of binding sites with G and and 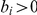 the affinity constant between 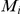 and G. The individual repressive impact of 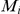 on 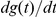 is as above defined by
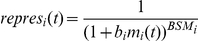
and the global repressive impact of all the miRNAs in MIR(G) on the rate of change 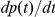 of protein P is given as above by
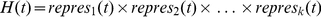
(7)


Let 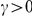 and 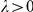 be the degradation rate and the translation rate for protein 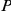. During the small time interval 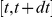, the concentraion of P molecules destroyed by natural degradation of P is 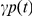, the concentration of G molecules committed to the translation of G is equal to 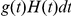, and hence the concentration of 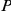 molecules generated by translation of 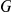 molecules is 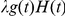. Thus the concentration 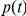 of protein P is driven by the following CKE, characteristic of the Transl.Inhib. architectures
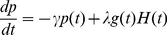
(8)


A probabilistic analysis justifying the preceding arguments is outlined in [12], [14]. Since 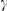 can be estimated from protein evolution recordings such as Western blots data, the CKE just derived depends on the 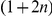 unknown positive parameters 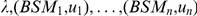.

### Parameter Estimation for Chemical Kinetics Models

For any given Transcr.Degr. or Transl.Inhib. architecture, an immediate challenge is to determine which parameter values must be injected in the corresponding CKE 6 or 8 in order to best fit our given sets of microarray data. We have developed and implemented innovative algorithms to compute these optimal parametric values.

For instance, consider the simplest Transcr.Degr. architecture model where there is only 1 transcription factor, which can either be a repressor or an activator. The number of parameters to be evaluated is 5 and after extrapolating the concentration data recorded by microarrays for both WT and GCNF-KO experiments on ES cells differentiation.

With the preceding notations, for Transcr.Degr. architectures, 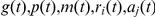 At a finite number of time values “t”, which we have extended to 19 instants by specific extrapolation, each one of our two microarray data sets (WT and GCNF-KO) provides observed values proportional to the concentrations of all the molecules involved in each one of our 5701 instances of Transcr.Degr. or Transl.Inhib. architectures. Hence after discretization of CKE 6 or of CKE 8 at 19 time instants, our two microarray data sets provide us with 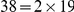 nonlinear algebraic equations (of high degree) involving the unknown parameters of the corresponding CKE.

For each Transcr.Degr. networks, the number 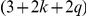 of unknown parameters remains between 5 and 15, since we have imposed 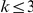 and 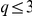.

For each Transl.Inhib. networks, the number 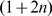 of unknown parameters remains between 3 and 7 since we have imposed 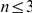.

Hence optimal parametrization of our 5701 genes interaction networks 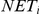 of types Transcr.Degr. or Transl.Inhib. was a numerical challenge since to parametrize each small network, we had to solve an overdetermined system of 38 algebraic equations of quite high degree involving between 3 and 15 unknowns. A natural mathematical approach is to solve the associated *highly non linear least squares* problem involving 38 equations and a number of unknowns inferior to 15.

There are no explicit solutions for such problems; moreover fast computing was essential here, since we had to implement the solutions to 5701 such nonlinear least squares problems.

This is a nonlinear optimization problem, since we want to select up to 15 *positive* parameters minimizing the sum of squares of 38 “residuals”, in order to optimize the quality of fit of the model under 38 equality constraints. We have of course tested several generic optimization approaches (see [15]–[18]) such as “gradient descent” and “genetic algorithms”. These last two well known optimization techniques turned out to require far too much computing time and were often unreliable due to their high dependence on initialization values.

So we had to develop and implement specific efficient algorithms dedicated to the parameterization of equations CKE 6 (or CKE 8) based on a combination of multi-scale searches for the 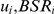 and the 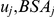 (or for the 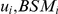) combined with constrained linear programming determination of the other parameters once the affinity constants and the numbers of binding sites have been tentatively fixed.

For CKE 6, the factors 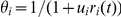 and 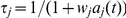 always lie within the range 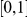, so we perform a grid search on the interval 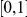 to explore the values of these factors at fixed key instants t, and then derive the associated potential values for 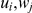. We also impose a moderate bound 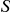 on the numbers of binding sites 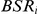 and 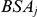. This of course generates a large set of potential values for the 2k parameters 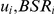 and the 2q parameters 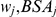. Once we fix tentative values for these 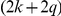 parameters, we can estimate the decay rate 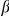, reaction rate 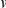, and transcription rate 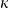 by solving a constrained linear programming problem in dimension 3 (see [14]).

For the parametrization of CKE 8, we have developed an analogous algorithm.

For both CKEs 6 and 8, our parametrization algorithm is quite fast and easily implemented. For each one of the 5701 small network selected above, the parametrization generated by our algorithm provides a good optimization for the quality of fit between CKE model and our two microarray data sets. To reach robust conclusions, our parametric modeling applies a “parameter parsimony” principle, selecting among models with high quality of fit to data, the models having the smallest number of parameters.

Our parametrized chemical kinetic modeling approach handles microarray data through *nonlinear parameterization algorithmics*, and clearly goes further than *linear* techniques such as Principal Components Analysis (PCA) or profiles correlation analysis. Our parsimonious modeling of small interaction networks, combined with the quality of fit evaluations outlined below, provides a powerful tool for microarray data analysis, complementing classical linear data mining techniques. Moreover, since our network models are either of Transcr.Degr. or Transl.Inhib. type, the miRNA-mRNA pairs we validate below are automatically assigned to one of the two basic repressive modalities Transcr.Degr. or Transl.Inhib.

### Validation of parametrized Transcr.Degr. and Transl.Inhib. architectures by Quality of Fit to data

To model the potentially interacting pairs (M,G) where M belong to our set of 266 miRNAs and G is one of the 10 key ES cell regulatory genes listed above, we have generated, as outlined above, a group of 5701 Small Interaction Networks which we denote 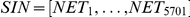. The group SIN comprises 364 Transcr.Degr. motifs and 5337 Transl.Inhib. motifs.

Each miRNA-mRNA pair (M,G) considered above was only retained when M targets G according to miRanda or Targetscan. Then we consider the family 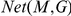 of all the networks which belong to the group SIN and include the pair (M,G). We will validate the pair (M,G) as a repressive miRNA-mRNA pair if and only if at least one of the networks in the subset Net(M,G) has been modeled by a parametrized CKE having a high enough *quality of fit* to our microarray data. We now need to precisely define this quality of fit.

Each specific network 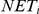 belonging to 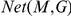 has been modeled by a chemical kinetic equation 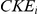 of type Transcr.Degr. or Transl.Inhib., and the parameters of 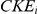 were computed to optimize the fit with our WT and GCNF-KO microarray data sets.

Call *D the downstream target* of 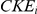. The molecule D is the gene G if 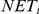 is of Transcr.Degr. type; if 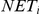 is of Transl.Inhib. type, D is the protein P produced by G. The concentrations D(t) of D are separately recorded by microarrays for WT ES cells and for GCNF-KO ES cells.

For each such experiment, the parametrized equation 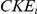 clearly generates model predicted values 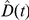 for the concentrations D(t) of the downstream target, by numerically solving the Ordinary Differential Equation (ODE) specified by 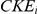. More precisely, the ODE 6 can be solved for the concentration 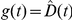 of 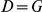, and the ODE 8 can be solved for the concentration 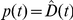 of P, since the recorded data provide values for all the 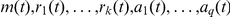 involved in the ODE 6 and for all the 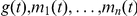 involved in the ODE 8.

To assess the quality of fit between the model predictions 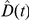 and the recorded microarray data 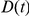, a natural criterion is the Relative Error of Prediction 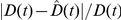. However this relative error of prediction becomes meaninglessly large whenever 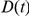 is close to zero. To avoid these spurious large values, we introduce the mean value 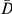 of 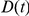 over all 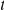, and we compute the *Smoothed Relative Error of Prediction* by
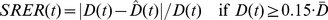
(9)

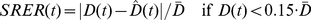
(10)Then we define the *global Error of Prediction* 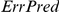 of the 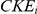 model by
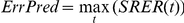
The error of prediction “ErrPred” is percentage valued and *quantifies the quality of fit* between the model 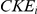 and the microarray data. Note that small values of ErrPred correspond to high quality of fit between model and data. Hence our parameters estimation algorithms were actually implemented to *select parameters minimizing ErrPred* for both WT and the GCNF-KO microarray data.

We will consider the network 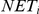 as *“validated’* if, for both the WT and the GCNF-KO microarray data, the global “error of prediction” 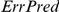 of the model 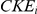 is less than 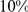.

To each miRNA-mRNA pair (M,G) considered above, we have associated the family 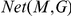 of all the networks 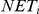 which include the pair (M,G) and belong to our group SIN of small networks. We will *validate the pair (M,G) as a repressive miRNA-mRNA pair* if and only if at least one of the networks 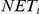 belonging to 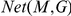 has been validated, as just outlined, by exhibiting small enough global errors of prediction.

Once an miRNA-mRNA pair (M,G) is actually validated, we can then rank all the validated networks 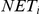 belonging to 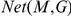 in decreasing order of reliability, which is equivalent to the increasing order for their errors of prediction ErrPred. To break ties between networks models with comparable ErrPred values, we apply a parameter parsimony principle and give priority to models with smaller numbers of parameters.

## Results

### Validated miRNA-mRNA Pairs of Transcr.Degr. Type

Our main results on validated miRNA-mRNA pairs of Transcr.Degr. type are summarized in Tables 1 and 2.

**Table 1:** Transcr.Degr. architectures: numbers of validated miRNA-mRNA pairs.

mRNA gene G	Oct4	Nanog	Sox2	Klf4	Esrrb	cMyc	Tbx3	Ezh1	Ezh2	Eed
# of miRNAs targeting G	19	2	29	44	10	12	18	51	29	25
# of validated miRNAs	**5**	0	9	2	0	12	18	20	12	7

**Table 2:** Transcr.Degr. architectures: the 85 validated miRNA-mRNA pairs.

miR-24; miR-103; miR-107; miR-186; miR-466	Oct4
miR-19a; miR-19b; miR-21; miR-129-3p; miR-182; miR-290; miR-292-5p;	Sox2
miR-339; miR-431	
miR-19a; miR-29b	Klf4
let-7b; let-7c; let-7f; let-7i; miR-96; miR-98; miR-135a; miR-135b;	cMyc
miR-182; miR-212; miR-340; miR-451	
miR-17-3p; miR-17-5p; miR-20a; miR-20b; miR-26a; miR-26b; miR-93;	Tbx3
miR-106b; miR-126-5p; miR-142-3p; miR-142-5p; miR-146; miR-146;	
miR-106a; miR-338; miR-448; miR-466; miR-469	
miR-15a; miR-15a; miR-15b; miR-16; miR-16; miR-22; miR-28;	Ezh1
miR-195; miR-195; miR-291a-3p; miR-301; miR-301; miR-302c; miR-323;	
miR-145; miR-183; miR-329; miR-342; miR-345; miR-449	
let-7a; let-7b; let-7c; let-7d; let-7e; let-7f; let-7g; let-7i;	Ezh2
miR-26a; miR-26b; miR-98; miR-98	
miR-1; miR-30a-3p; miR-101a; miR-301; miR-323; miR-337; miR-34b	Eed

We had initially constructed 364 small networks of Transcr.Degr. type, for which the mRNA downstream targets belonged to one of the 3 following sets of ES cell regulatory genes: the self-renewal regulators (Sox2, Klf4, cMyc, Tbx3, Esrrb), the differentiation inhibitors (Ezh1, Ezh2, Eed), and the differentiation regulators Oct4 and Nanog.

For the 2 downstream targets Oct4 and Nanog, as seen in Figure 2, the proteins produced by Oct4, Nanog, Sox2 are transcription activators [8], [11], GCNF is a key transcription repressor, and the potential miRNAs transcription repressors included 19 miRNAs for Oct4 and 2 miRNAs for Nanog. We had generated potential lists of upstream transcription repressors for Oct4 and for Nanog by combining each one of these 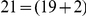 miRNAs with GCNF and with one of the 7 short lists (Oct4), (Nanog), (Sox2), (Oct4, Nanog), (Oct4, Sox2), (Nanog, Sox2), (Oct4, Nanog, Sox2).

After parametrization and validation of these Transcr.Degr. models repressing the downstream target Oct4, only 5 repressing pairs (miRNA,Oct4) were validated by high quality of fit to data, and they involved the five miRNAs (miR-24, miR-103, miR-107, miR-186, miR-466). Each one of the 5 corresponding validated Transcr.Degr. architectures combined one of these 5 miRNA repressors of Oct4 with the 3 transcriptional repressors (GCNF, Oct4, Nanog).

**Figure 4:**
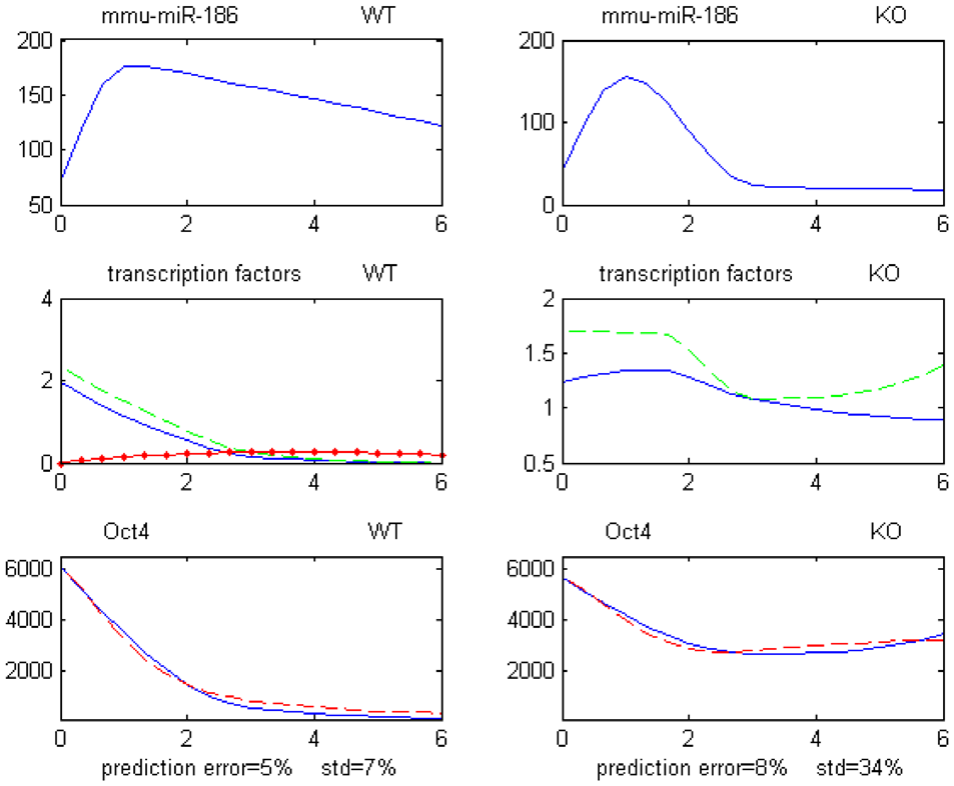
Example of small network of Transcr.Degr. type repressing mRNA Oct4. All expression profiles are over days 0–6. Top profiles: miRNA miR-186 for WT and GCNF-KO data. Middle profiles: transcription factors of Oct4 for WT and GCNF-KO data. Blue solid line = protein Oct4. Green dash line = protein Nanog. Red dotted-solid line = protein GCNF. Bottom profiles: mRNA Oct4 for WT and GCNF-KO data. Blue line = recorded levels. Red dash line = predicted levels. “prediction error” is the model global relative error of prediction; std is the relative standard deviation of recorded levels.

Figure 4 displays expression profiles corresponding to one of these 5 validated Transcr.Degr. model, with upstream transcription repressors mmu-miR-186, GCNF, Oct4, Nanog, and with transcription activators (protein Oct4, protein Nanog).

For the 2 downstream targets Nanog and Esrrb, as reported in Table 1, there were no validated repressing pair (miRNA, Nanog) or (miRNA, Esrrb) of Transcr.Degr. type. However, our datasets of recorded microarray data contained only 266 known miRNAs, so our modeling could not include a small group of miRNAs known to be potentially targeting Nanog or Esrrb, but for which we had no recorded microarray data.

We omit details (see [14]) and refer to Tables 1 and 2 for the other 6 downstream targets (Klf4, cMyc, Tbx3, Ezh1, Ezh2, Eed). Over all our initial 364 models of Transcr.Degr. type, only 88 models were validated after parametric modeling. In Table 2, we list all the 85 *validated* miRNA-mRNA pairs of Transcr.Degr. type repressing one of the 10 downstream targets (Oct4, Nanog, Sox2, Klf4, cMyc, Tbx3, Esrrb, Ezh1, Ezh2, Eed).

### Validated miRNA-mRNA Pairs of Transl.Inhib. Type

We had initially generated a list of 5337 small networks of Transl.Inhib. type potentially inhibiting the translation of one of the 4 downstream targets (Oct4, Nanog, Sox2, GCNF). Each one of these 5337 networks involved at least 1 and at most 3 of the 133 miRNAs targeting one of the 4 downstream genes (Oct4, Nanog, Sox2, GCNF).

After parametric modeling of these 5337 networks, and validation by requesting high quality of fit to data, we have validated only 24 miRNAs as translation inhibitors repressing one of these 4 downstream proteins Oct4, Nanog, Sox2, GCNF. These results are summarized in In Tables 3 and 4.

**Table 3:** Transl.Inhib. architectures: numbers of validated miRNA-mRNA pairs.

mRNA gene G	Oct4	Nanog	Sox2	GCNF
# of miRNAs targeting G	19	2	29	83
# of validated miRNAs	13	0	0	11

**Table 4:** Transl.Inhib. architectures: the 24 validated miRNA-mRNA pairs.

miR-103; miR-107; miR-138; miR-186; miR-218; miR-24; miR-324-5p;	Oct4
miR-337; miR-338; miR-369-5p; miR-466; miR-484; miR-542-3p	
let-7e; let-7g; miR-10a; miR-10b; miR-23b; miR-30c;	GCNF
miR-124a; miR-181b; miR-214; miR-351; miR-382	

Among the 19 miRNAs targeting Oct4, only 13 were validated as inhibiting the translation of Oct4. Each one of these 13 miRNA inhibitors of Oct4 was included in several of the 51 validated Transl.Inhib. architectures repressing Oct4. Each one of these 51 validated architectures involved a group of 3 miRNAs inhibiting the translation of Oct4.

**Figure 5:**
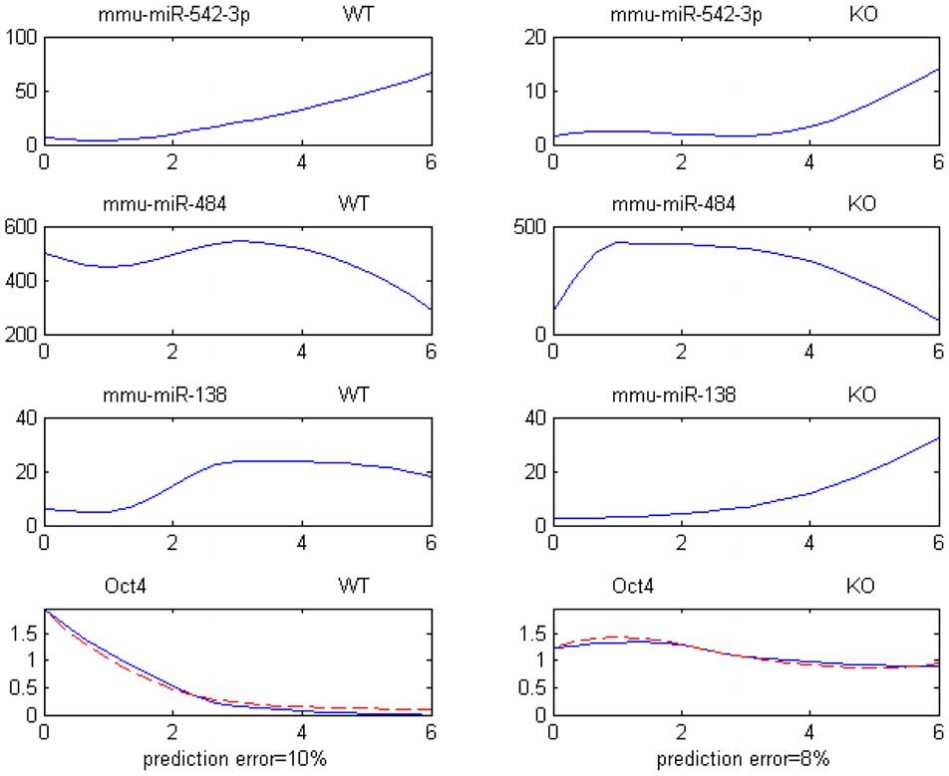
Example of Transl.Inhib. architecture repressing Oct4. All expression profiles are over days 0–6. Upper 6 profiles: miRNAs miR-542-3p, miR-464 and miR-138 for WT and GCNF-KO. Bottom 2 profiles: Blue line = recorded level. Red dash line = predicted levels.

Figure 5 displays the expression profiles for one example of validated network inhibiting the translation of Oct4 through 3 upstream miRNA repressors (mmu-miR-542-3p, mmu-miR-484, mmu-miR-138).

Among the 11 validated miRNAs inhibiting the translation of GCNF, we find no miRNA in the high-low class HL, 4 miRNAs in the low-high class LH, and 7 miRNAs in the transient class TR. This result agrees quite well with [5], which indicates that miRNAs of class HL do not repress GCNF but that some miRNAs of class LH may repress GCNF.

Among the 83 miRNAs targeting GCNF, we have validated only 11 miRNAs inhibiting the translation of GCNF. For the GCNF protein, we naturally only have profile data for the WT ES cells since GCNF vanishes in GCNF-KO data. The number of data points for GCNF data hence half of the data points available for other proteins. The Transl.Inhib. architectures potentially repressing GCNF were thus restricted to include only one miRNA targeting GCNF, in order to restrict the number of model parameters.

Finally, among the 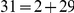 miRNAs potentially targeting Nanog or Sox2, none could be validated within a translation inhibiting pair of the form (miRNA, Nanog) or (miRNA, Sox2). Let us clarify further this situation. Since the Nanog protein is potentially repressed by only 2 miRNAs (see [4]), we could include one or both of these miRNAs in only 3 potential Transl.Degr. architectures. The fact that none of these 3 networks could be validated by high quality of fit to data is not surprising, since the rate of attrition due to model validation was fairly high in general.

For the Sox2 protein, we had a choice of 29 miRNAs potentially targeting Sox2, and hence a large set of 1653 Transl.Inhib. models to parametrize. For all these models, after parametric fitting to both WT and GCNF-KO data,the estimated translation rate 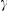 was almost zero. So no matter what upstream miRNAs were selected, the modeling prediction for Sox2 remained the same. We have traced this effect to the modeling assumption that the degradation rates of the downstream protein remains the same for both WT and GCNF-KO experiments. But our Western Blots data indicate that the Sox2 protein degradation rate in WT ES cells seems to be larger than in GCNF-KO ES cells.

Note that the half-life of a protein like Sox2 may quite possibly change in different cell cultures; for instance, protein Oct4 has a half-life of 90 minutes in undifferentiated P19 cells [19] and of 6 to 8 hours in NIH3T3 cells transfected with wild type Oct4 [20].

If we now generate parameterized models of the same translation-inhibition architectures, but with the new assumption that the half-life of protein Sox2 is different for WT cells and for GCNF-KO cells, then we obtain much better model predictions. The estimated half-life of protein Sox2 is then 20.5 hours for WT cells, and 64.5 hours for GCNF-KO cells. We have left this question opened for the moment, until further experiments help us generate a concrete conclusion on the possibly distinct values for the half-life of protein Sox2 in WT cells and in GCNF-KO cells.

## Discussion

Our goal was to analyze the regulatory role of miRNAs in ES cells differentiation, on the basis of two sets of microarray data, recorded for WT and for GCNF-KO ES cells.

We have modeled, by 2 basic chemical kinetic equations 6 and 6, the 2 main functions of miRNAs in post-transcriptional down-regulation of genes expression. These CKEs correspond to 2 formal types (Transcr.Degr. and Transl.Inhib.) of interaction architectures between miRNA, mRNA, and associated proteins.

Starting with the 266 miRNAs recorded in our microarray data, we have focused on their potential repressive impact on 11 key regulatory mRNA genes of ES cells differention, namely (Oct4, Nanog, Sox2, Klf4, cMyc, Tbx3, Esrrb, Ezh1, Ezh2, Eed) for the Transcription Degradation modality and the (Oct4, Nanog, Sox2, GCNF) proteins for the Translation Inhibition modality.

We have retained as potentially interacting only the miRNA-mRNA pairs (M,G) in which M targets G according to miRanda or Targetscan. Each such miRNA-mRNA pair was naturally imbedded within one of 5701 small genes interaction networks, namely 364 networks of Transcr.Degr. type and 5337 networks of Transl.Inhib. type. We have developed an innovative algorithmic approach to parametrize the corresponding 5701 chemical kinetics equations by optimizing their quality of fit to our two sets of microarray data. Our numerical algorithm solves efficiently a nonlinear least squares fitting of 38 high degree algebraic equations involving between 3 and 15 unknown parameters. Each one of the 5701 parametrized CKE we thus obtained was then tested for the accuracy with which the CKE could predict the expression profiles of its downstream mRNA gene target (or protein target), for comparison with our WT and GCNF-KO microarray data. The corresponding small network was considered as valid if and only if the predicted and recorded expression profiles of these down stream target were well matched, with a relative error of prediction inferior to 10%.

The list VAL of interaction networks which were validated by high quality of fit to data was naturally much smaller than our initial list of 5701 small networks modeling potential miRNA-mRNA interactions. We have then considered that a potential miRNA-mRNA pair was validated if and only if it had been imbedded in at least one of the networks belonging to VAL. We have thus determined 109 “model validated” miRNA-mRNA pairs, namely 85 pairs interacting by Transcription Degradation of the mRNA target, and 24 pairs interacting by inhibiting the translation of their gene target into protein. These results, summarized in Tables 1, 2, 3, 4, should help to circumscribe further experimental gene expression analyzes on miRNA-mRNA pairs.

For any given mRNA gene G in our 11 key genes regulating of ES cells differentiation, our results provide very short lists of model validated miRNAs repressing G. A typical experimental validation will be to first pick two such miRNA candidates, 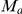 degrading the transcription of G, and 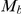 inhibiting the translation of G. As in (see Methods in [4]), one could transfect one set of wild-type ES cells with precursor miRNA (pre-miRNA) oligomers associated to 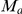 and similarly transfect another set of ES cells with 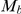. Recording the expression levels of mRNA gene G should enable the comparison of the two rates of change of G after a short transfection time, for both sets of ES cells, considering that, in such a short time, the expression levels for the transcription factors of G are not yet likely to be influenced to any great extent. Prediction by parameterized modeling should help the quantitative interpretation of the experimental recordings and enable concrete conclusions on the interactive parts played by 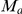 and 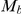.

When fairly comprehensive knowledge of the transcriptional factors for a specific mRNA gene G is available, and for any new set of microarray data, we can implement our automated modeling and validation of miRNA-mRNA pairs involved in transcription-degradation architecture just as above. A key facilitating point would be the availability of associated proteins expression levels, which are difficult to measure simultaneously for a large set of proteins. But we can still use our Transcr.Degr. CKEs to determine whether the proteins with actually recorded expression levels are indeed transcriptional factors for the mRNA gene G.
